# Ethnoveterinary medicines in four districts of Jimma zone, Ethiopia: cross sectional survey for plant species and mode of use

**DOI:** 10.1186/1746-6148-10-76

**Published:** 2014-03-28

**Authors:** Yared Yigezu, Demissew Berihun Haile, Wubeante Yenet Ayen

**Affiliations:** 1 Pharmaceuticals Fund and Supply Agency, Addis Ababa, Ethiopia; 2 Department of Pharmacy, Mizan- Tepi University, P.O. Box 260, Mizan, Ethiopia; 3 Department of Pharmaceutics, Pharmacy School, Jimma University, Jimma, Ethiopia

**Keywords:** Ethnoveterinary, Medicines, Plant species, Mode of use, Jimma, Ethiopia

## Abstract

**Background:**

Traditional medicines have been used for nearly 90% of livestock populations in Ethiopia where complimentary remedies are required to the modern health care system. All plants with pharmacological activity complimentarily prescribed as best choice against livestock diseases. A community based cross - sectional survey was conducted to investigate ethno-veterinary knowledge and practices of study area by purposive sampling techniques. The data from respondents were collected through face-to face interview using pre-tested semi-structured questionnaires, which was further accompanied by field observations of the medicinal plants. The vast majority of the statistics were analyzed descriptively by SPSS 16 Windows version to extrapolate our findings in ethno-botanical knowledge.

**Results:**

In the study, a total of 74 species of ethnoveterinary medicinal plant species from 31 families have been identified for treating 22 different livestock ailments. The three families: Asteraceae, Cucurbitaceae and Solanaceae make up larger proportion of reported medicinal plants which accounted for 10.41%, 8.33% and 6.25%, respectively. Of reported medicinal plants, 16.7% informant consensus was recorded for the species *Croton macrostachyus* Del., 10.7% for *Nicotiana tabacum* L. and 9.5% for *Olea capensis* L.Subsp. *macrocarpa* (C.H. Wright) I.Verd. in treatment of one or more veterinary ailments. The greater varieties of medicinal plant species that accounted for 28.2% were used against management of blackleg which was common livestock diseases in the study area. The findings showed, trees accounted for 43.24%, followed by shrubs (33.78%) and herbs (14.86%). Eighty one percent of medicinal plants reported by respondents were collected from wild habitats, and leaves reported to be used by 68% of the informants for ethnoveterinary medicines preparations. The preparations were applied through different routes of administration; oral administration accounted for (76.2%), followed by application of topical (9.53%) and nasal (5.19%).

**Conclusions:**

Ethnoveterinary practices significantly suggested to play greater roles in livestock health care as an alternative or integral part of modern veterinary practices. The traditional knowledge in treatment of livestock diseases of the study districts needs further scientific evaluations by phytochemical and antimicrobial experimentation to determine safety, efficacy, mode of delivery, drug development and dosage in pharmacological laboratory.

## Background

Ethnoveterinary medicine, defined as the “application of veterinary folk knowledge, theory and practice to treat ailments of livestock,” has been the focus of several studies in the last few decades [1]. The current rich and resourceful traditional knowledge of ethnoveterinary have been transferred to generations by words of mouth only [2]. A large number of farmers rely on a range of ethnoveterinary knowledge to keep their livestock healthy and have been used for preventing and treating livestock ailments for several generations. Diversity of plant species which have pharmacological activities were identified so far and the active ingredients are extracted mainly from the root, stem, and leaf parts that processed to administer through appropriate routes [3]. They traditionally learned to diagnose clinical features of endemic animal diseases by the use of traditional equipments like bamboo syringes, stone tourniquets, animal horn products, squashing with their hands, and wood forceps to determine dosage, and prepare remedies from local medicinal plants. Treatment by contemporary veterinary medicine has, in these days, been out of the reach of the ordinary farmers often due to high cost of drugs and none coverage [4,5]. The farmers have subsequently explored many ethno-botanical products in treating livestock diseases [6]. With a view of those traditional practices, the world health organization (WHO) declared the important roles of ethno-botanical products in veterinary and human medicines in the Alma-Ata proclamation in 1978. Additionally, WHO also stated that the use of these natural products in control of animal and human diseases are considerably effective [7]. In 1996, the American Veterinary Medicine Association also officially stated that botanical products are safely used in compliment of National Intervention Program of livestock diseases [8].

Many studies assured that farmers and pastoralists in several countries such in West Java, Indonesia [9], Mexico, Nigeria [10], Ormaland, Kenya [11], Zimbabwe [12], South Africa [13], China [14], Pakistan [15] and India [16] widely use medicinal plants in conservation of the health of livestock. Similarly, across all regions of Ethiopia since long time have been using ethnoveterinary knowledge to treat livestock disease for thousands of generations [4,5,17-22]. It is estimated that up to 90% of current livestock diseases are managed through the use of traditional medicines [23]. The livestock production in Ethiopia has been beset by many problems which mainly include poor nutrition, poor management and diseases; whilst in recent times livestock diseases cause more economic losses [4].

Practices in ethnoveterinary medicine solely depend on the collective memories of just a few practitioners within communities, being it is not such a common knowledge for everybody. However, such increase in ethnoveterinary knowledge, which has been transferred verbally from one generation to the next from immemorial time, is currently in a danger of being lost whenever a traditional veterinary practitioner passes away without conveying his traditional medicinal plants knowledge [24].

Despite these facts, very little efforts have so far been made to reverse the trends and promote skills effectively throughout modern time courses. Few ethnoveterinary surveys have been conducted in Ethiopia, such as Borena pastoralists [5], different studies in two localities of Tigray [4,20], Fentale, Esternshowa [21], Boosat, Welenchetti [17], Bale Mountains National Park [18], and Gilgel Ghibe [19], but only Goma district in Jimma zone [25] to document the well known ethno-botanical products used in animal health care practices. Therefore, this study was conducted to identify and scientifically document plants applied in ethnoveterinary practices along with livestock ailments treated by farmers in four districts of Jimma zone.

## Methods

### Study design

A community based cross sectional study was conducted to investigate ethnoveterinary knowledge and practices of four districts of Jimma zone, Southwestern, Ethiopia. Specimen Vouchers were given on spot for each plant species and later identified using taxonomic keys in the relevant volumes of the flora of Ethiopia and Eritrea in National Herbarium. Additionally, botanical taxonomists also made comparisons visually with authenticated plant specimens kept at national herbarium (ETH) of Addis Ababa University. Finally, the findings of the study specimen’s vouchers have been deposited at the national herbarium (AAU).

### Study area profiles

Jimma zone is one of the thirteen zones of Oromia regional state which geographically lies at southwestern part of Ethiopia. Jimma Town is the capital of the zone that is 345 km far away from Addis Ababa, capital city of Ethiopia. It covers a total surface area of 19,305.5 km^2^. According to the 2007 Population and Housing Census of Ethiopia, the total population of Jimma zone was 2,486,155. From the total population in the zone, 2,204,225 (88.66%) is the rural population, which directly depends on agricultural activities for domestic use and exchange of commodities with urban residents. The zone bordered in Northwest by Illubabor, in East by Wellega and in West by Shewa zones as well as in south by Southern Nations and Nationalities People’s Regional state.

In general, topographical features elevation varies from 1000 to 3360 m above sea level with average maximum and minimum temperatures in range of 25–30°C and 7–12°C, respectively. Annual rainfall of the zone is one of the highest in the country reaching up to 1200–2800 mm per year. The predominant economic activities involve mixed farming, which broadly includes cultivation of cereal crops, cash crops including primarily coffee and production of livestock.

Jimma zone has an estimated of 1,718,284 heads of cattle, 466,154 sheep, 194,677 goats, 74,774 horses, 40,555 donkeys and 30,541 mules populations [26]. According to the Zone agricultural and rural development office there are about 42 animal health clinics in the zone which are being run by 84 animal health professionals (5 Doctors of Veterinary Medicine, 12 animal health assistants and 67 animal health technicians) and the current animal health services coverage reaches 25% (Un-published data, Jimma Zone agriculture and rural development office, 2002).

**Figure 1:**
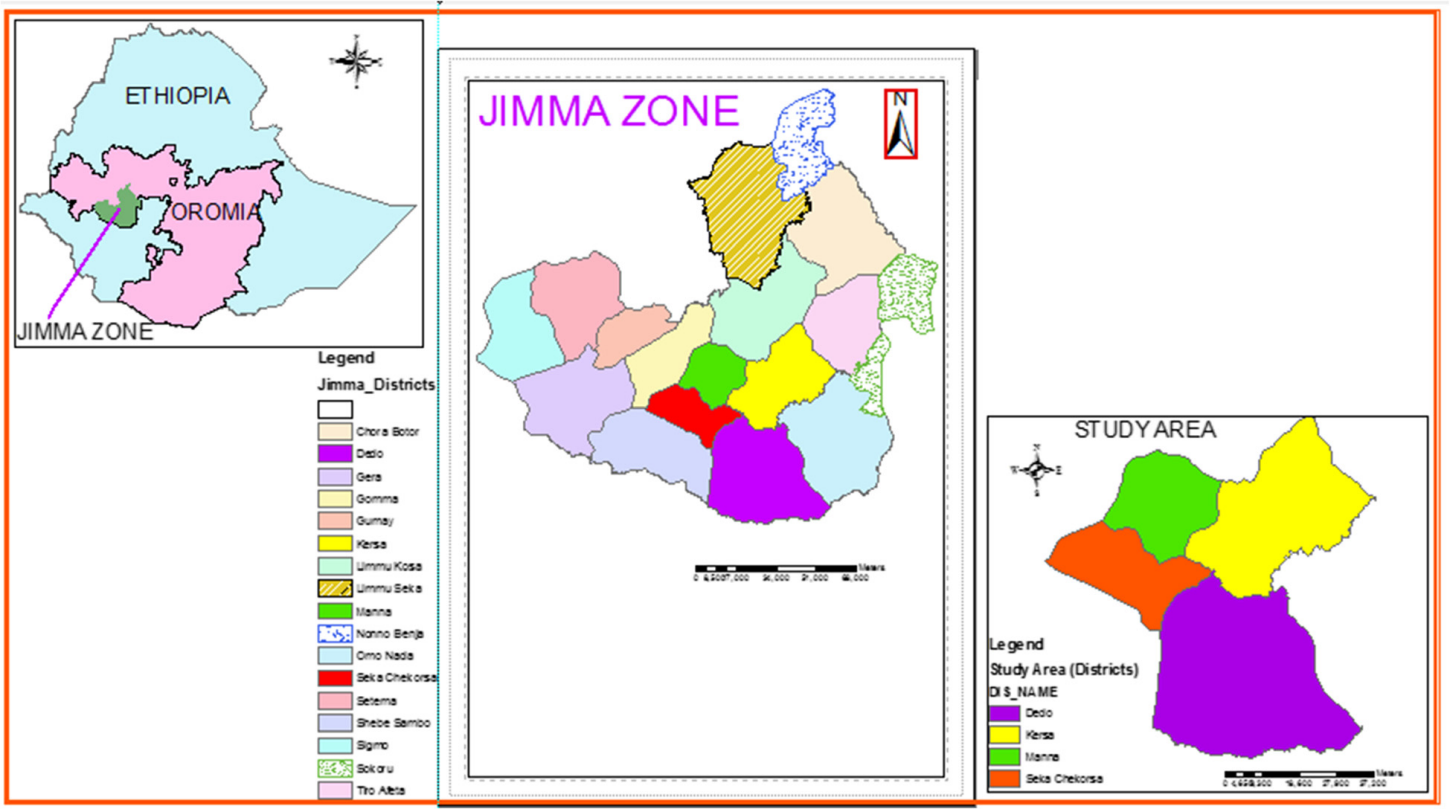
Map showing geography of study districts.

The present survey was conducted in 4 districts of Jimma zone: namely, Seka chekorsa 20 km in Southeast, Dedo 20 km in South, Mena 22 km in Northeast and Kersa 18 km in Northwest, of Jimma Town. According to recent zoning system, the zone is divided into 17 districts and 22 urban centers (Figure 1).

### Selection for study districts and participants

The zone where the study conducted was divided into 17 strata based on their recent administrative districts, of these, 4 districts were purposefully selected as these communities highly depend on traditional healings and possess many skills acquired from foreparents. Besides considering their wide use of traditional ethno-botanical products that meet our purpose, the study were limited to these four districts to increase feasibility of the study. Selection of informants was performed conforming to Martin [27] who stated that when recording indigenous knowledge controlled by ethnobotanical healers or by certain social groups the choice of key informant is vital. For our study units, a total of 84 informants (79 male and 5 female): 26 from Kersa, 27 from Dedo, 15 from Seka chekorsa, and 16 from Mena districts were purposively selected with help of local people and governmental bodies, who are considered to be key informants. The selected healers were well known in the community due to their long practice in providing services related to veterinary medicinal plants. During data collection, preliminary discussion was individually held with the key informants through assistance of local elders to elaborate the objective of the study. That was done to clarify the purpose and persuade the respondents to provide reliable information without suspicion and to explain them that their cooperation is a valuable contribution to the documentation of the traditional medicinal plants of the districts.

### Data collection

Data collection was conducted over a period of 4 weeks, from March to April, 2007. The interview was made by using semi-structured questionnaires in English version that was translated into Oromic local language. Necessary information with view to local name of ethnoveterinary medicinal plants, methods of preparation, diseases treated with traditional remedies, route of administration, ingredients involved, habitat where plant adapted to, and parts of the medicinal plants involved were recorded. Next, the researchers with help of traditional practitioners made field visits to take samples of each medicinal plant in respective area.

### Data quality assurance

With purpose of data quality assurance during interview, each informant was contacted at least two to three times for the same ideas and the validity of the information was proved and recorded. In case, the idea of the informant deviated from the original information, it was rejected as it was regarded irrelevant information. Only the relevant data were taken into account and statistically analyzed. Further, the data quality was ensured through training of data collectors, pretesting of instruments, checking of missing data, data cleaning and double entry, and careful data analysis.

### Statistical analysis

A descriptive statistics, mainly mean ± standard error of mean (SEM) for normally distributed continuous variables, median with lower quartile (Q1) and upper quartile (Q3) for non-normally distributed continuous variables, and frequency analysis were performed to summarize the ethno-botanical product data reported from respondents. In this study, the Kolmogorov–Smyrnov test was used to determine the Gaussian distribution of the study variables. The data were subjected to statistical analysis using statistical package for social sciences (SPSS) software windows version 16.0.

### Ethical statement

This study was approved by the Institutional Review Board, Jimma University. Accordingly, the confidentiality of traditional property owners was completely maintained in processing our data. During data collection an effort was verbally made to encourage the traditional healers of livestock in such a way that their cooperation is of great benefit to the country and also revelation of their knowledge of medicinal plants will not in any way interfere with the continued practice of their art. All data from this study weren’t shared with third party out of researchers. Additionally, informed consent was obtained from the participants to ensure their willingness.

## Results

### Socio demographic characteristics of the informants

In this survey, a total of 84 informants of which 79 (94.05%) was male and 5 (5.95%) was female respondents were involved. The age of the respondents distributed from 20 to 80 years with mean of 48 ± 12.3 years but their experiences in livestock farming varied from 5 to 60 years with mean of 22 ± 15.3 years. Educational status analysis showed that, 47 (55.95%) of the respondents were illiterates, 17 (20.24%) of them were able to write and read, while the remaining 14 (16.67%) and 6 (7.14%) were in between grade 4 to 8 and 9 to 12, respectively.

### Medicinal plants and their applications

In our investigation, a total of 74 plant species (14 from Seka chekorsa, 19 from Kersa, 20 from Dedo and 21 from Manna district) were used against 22 types of livestock diseases and to increase livestock outputs. Those plants were botanically distributed across 54 genera and 31 families (Tables 1 and 2). Data from our study suggested that the highest number of plant species was found in Asteraceae family with (10.41%) followed by Cucurbitaceae (8.33%) and Solanaceae (6.25%). Of the total, 12 (16.22%) species were found to have been commonly used everywhere in four districts for similar or different veterinary purposes.

**Table 1:** Plants used for treatment of veterinary ailments with two or more species prescription by farmers in four districts of Jimma zone

**Scientific name (Family)**	**Vermicular name**	**Method of preparation (Ethno formulation)**	**Route**	**Claimed use**	**Voucher no.**
*Momordica foetida* Schumach.(Cucurbitaceae)	Minaan bofa (AO)	Root and leaves are pounded, mixed with water liquid extract are given to donkey (2 cups/dose)	Nasal, oral	Abdominal colic	YY02
*Ocimum lamiifolium* Benth.(Lamiaceae)	Damakase (AM)				YY06
*Ricinus communis* L.(Euphorbiaceae)	Qobo (AM)	All are pounded together in hot water to make decoction and given to cattle	Oral	Black leg	YY08
*Dieliptera acanthaceae* C.B.el (Acanthaceae)	Togo (AO)				YY33
*Ocimum lamiifolium* Benth.(Lamiaceae)	Damakase (AM)				YY06
*Leonotiso cymifolia* (Burm.F)(Lamiaceae)	Raskimir (AM)				YY59
*Brucea antidysenterica* J.F. Mill.(Simaroubaceae)	Aballo (AM)				YY16
*Ekebergia capensis* Sparm.(Meliaceae)	Sombo (AO)				YY70
*Allium sativum* L. (Alliaceae)	Nech shinkurt (AM)	The first 2 are powdered together and given to cattle, after half hour, concoction made from the remaining ingredients is given for cattle (1/2 cups/dose)	Oral	Black leg	YY11
*Lepidium sativum* L.(Brassicaceae)	Fexo (AO)				YY15
*Echinops kebericho* Mesfin (Asteraceae)	Kebericho (AO)				YY34
*Clematis hirsute* perr. Guill (Ranunculaceae)	Nechazo (AM)				YY69
*Gouania longispicata* Engl. (Rhamnaceae)	Omochisa (AO)				YY62
*Acmella caulirhiza* Del. (Asteraceae)	Yemdir beriberi (AM)	Powder them separately then, add to hot water to make a decoction.	Oral	Stimulate lactation	YY45
*Withania somnifera* (L.) Dunal (Solanaceae)	Gizawa (AM)				YY24
*Senna didymobotrya* (Fresen.) Irwin & Barneby (Fabaceae)	Sanaa maki (AO)	First give fresh milk, then pulverized leaves with soap	Oral	African horse sickness	YY38
*Croton macrostachyus* Del (Euphorbiaceae)	Bisana (Am)				YY10
*Viscum tuberculatum* A.Rich. (Viscaceae)	Harmo (AO)				YY59
*Lepidium sativum* L.(Brassicaceae)	Fexo (AO)	Leaves are pulverized filtered and given to cattle.	Oral	Black leg	YY15
*Croton macrostachyus* Del (Euphorbiaceae)	Bisanna (AM)				YY10
*Ocimum lamiifolium* Benth.(Lamiaceae)	Damakase (AM)	Leaves are pounded and extract is given to cattle (2 teaspoons/dose)	Nasal	Trypanosomiasis	YY06
*Croton macrostachyus* Del (Euphorbiaceae)	Bisanna (AM)				YY10
*Vernonia amygdalina* Del.(Asteraceae)	Ebicha (AO)	All leaves are pounded, mixed with water, squeezed, filtered and given to cattle (1 cup/dose)	Oral	Diarrhea, Black leg, and Anthelmintic	YY32
*Croton macrostachyus* Del (Euphorbiaceae)	Bisana (AM)				YY10
*Nuxia congesta* R.Br. ex Fresen.(Loganiaceae)	Chocho (AO)				YY44
*Justicia schimperiana* (Hochst.exNees) T. Anders (Acanthaceae)	Sensel (AM)				YY30
*Euphorbia schimperiana* Scheele (Euphorbiaceae)	Binebisha (Yem)				YY18
*Lepidium sativum* L.(Brassicaceae)	Fexo (AM)	Seeds are powdered and mixed with pounded leaves then given to cattle (1/2 L/dose)	Oral, topical	Diarrhea, Skin infection	YY15
*Calpurnia aurea* (Aiton) Benth.(Fabaceae)	Digita (AM)				YY03
*Fagaropsis angoleusis (*Rutaceae)	Sigilu (AO)	Leaves are powdered together, soaked, squeezed; liquid extract is given to cattle (1 cup/dose)	Oral	Anthrax	YY35
*Clematis longicauda* Steud (Ranunculaceae)	Nech hareg (AM)				YY19
*Solanum marginatum* L.f.(Solanaceae)	Hiddi (AO)	Root and leaves are pounded together by adding water, filtered and given to live stock	Oral	Black leg	YY26
*Curcumis ficifolius* A. Rich. (Cucurbitaceae)	Faca’ aa (AO)				YY40
*Ocimum lamiifolium* Benth.(Lamiaceae)	Damakase (AM)	Leaves and fruit are pounded together; little water is added, filtered and given to cattle.	Oral	Black leg, Respiratory manifestations	YY06
*Calpurnia aurea* (Aiton) Benth.(Fabaceae)	Ceekaa (AO)				YY03
*Solanum marginatum* L.f.(Solanaceae)	Hiddi (AO)				YY26
*Senna didymobotarya* Fresen.(Fabaceae)	Sanaa maki (AO)				YY38
*Echinops kebericho* Mesfin (Asteraceae)	Kebericho (AO)	Root is powdered and mixed with pulverized leaves to make decoction	Oral	Black leg, Respiratory manifestations, Liver disease	YY34
*Nicotiana tabacum* L.(Solanaceae)	Tambo (AM)				YY13
*Vernonia amygdalina* Del.(Asteraceae)	Ebicha (AO)	All are pounded, mixed with water, squeezed, filtered and given to cattle.	Oral	Diarrhea, Respiratory manifestations	YY32
*Croton macrostachyus* Del (Euphorbiaceae)	Bisana (Am)				YY10
*Nicotiana tabacum* L.(Solanaceae)	Tambo (AM)				YY13
*Calpurnia aurea* (Aiton) Benth.(Fabaceae)	Ceekaa (AO)				
*Solanum marginatum* L.f.(Solanaceae)	Hiddi (AO)				YY26
*Solanum marginatum* L.f.(Solanaceae)	Hiddi (AO)	All are pounded together to make a decoction, filtered and given to cattle.	Oral	Black leg	YY26
*Nicotiana tabacum* L.(Solanaceae)	Tambo (AO)				YY13
*Clematis longicauda* (Ranunculaceae)	Fitti (AO)				YY23
*Viscum tuberculatum* A.Rich.(Viscaceae)	Harmo (AO)				YY59
*Calpurnia aurea* (Aiton) Benth.(Fabaceae)	Ceekaa (AO)	A decoction is prepared, filter and given to cattle.	Oral	Black leg	YY03
*Senna didymobotrya* (Fresen.) Irwin & Barneby (Fabaceae)	Sanaamaki (AO)				YY38
*Fagropsis angoleusis (*Rutaceae)	Sigilu (AO)	All leaves are pounded together to make a decoction, then filtered and given to cattle.	Oral	Respiratory manifestations, Black leg	YY35
*Senna didymobotrya* (Fresen.) Irwin &Barneby(Fabaceae)	Sanaamaki (AO)				YY38
*Clausena anisata* Benth (Rutaceae)*.*	Urgesa (AO)				YY61
*Clematis longicauda* Steud (Ranunculceae)	Nechharegi (AM)				YY19
Not Botanically Classified (NBC)	Kedida (AO)				YY45
*Vernonia sp.* (Asteraceae)	Omboroko (AO)				YY01
*Datura stramonium* L. (Solanaceae)	Asangira (AO)				YY47
*Senna didymobotrya* (Fresen.) Irwin & Barneby (Fabaceae)	Sanaamaki (AO)	Leaves are pounded together; the fruit extract is added, filtered and given to cattle.	Oral	Black leg	YY38
*Helinus mystacinus* (Ait.) E. Mey. ex Steud. (Rhamnaceae)	Omochesa (AO)				YY29
*Citrus Sinensis* (L.) Osb. (Rutaceae)	Birtukan (AM)				YY51
*Vernonia auriculifera* Hiern.(Asteraceae)	Rejii (AO)	All leaves are pounded together; mixed with 1 litter of water and given to livestock (1 L/dose)	Oral	Internal parasite	YY72
*Maytenus senegalensis* (Lam.) Excell (Celastraceae)	Kombolcha (AO)				YY54
*Catha edulis* (Vahl) Forssk.ex Endl.(Celastraceae)	Chat (AM)				YY20
*Momordica foetida* Schumach.(Cucurbitaceae)	Minaan bofa (AO)				YY02

*AO*, Afan Oromo; *AM*, Amharic.

**Table 2:** Plants used for treatment of veterinary ailments with single species prescription by farmers in four districts of Jimma zone

**Scientific name (Family)**	**Vernacular name**	**Method of preparation (Ethno formulation)**	**Route**	**Claimed use**	**Voucher no.**
*Fagaropsis angoleusis* (Rutaceae)	Siglu (AO)	Fresh leaves are crushed, water and salt are added, then given to cattle (0.5 L/dose)	Oral	Liver fluke and fattening of cattle	YY35
*Vernonia amygdalina* Del.(Asteraceae)	Ebicha (AO)	Fresh leaves are pounded with salt, then water is added and given to cow for 3 days in the morning	Oral	To improve milk production in cows	YY32
*Verbascum sinaiticum* Benth. (Scrophulariaceae)	Suffi (AO)	Fresh leaves are pounded after adding water, then squeezed and given to cattle	Oral	Skin infection	YY54
*Dodonaea angustifolia* L.F.(Sapindaceae)	Ittacha(AO)	Fresh leaves are crushed, water is added and given to livestock for 1–2 days (2 cups/dose)	Oral	Bloat	YY36
*Allophylus macrobotryls* Gilg. (Sapindaceae)	Gursedi (AO)	Fresh leaves are crushed and mixed with water and given to cattle (1 cup/dose)	Oral	Internal parasite	YY62
*Pentas schimperiana* (A.Rich)Vatke (Rubiaceae)	Surma (AO)	Leaves and stems crushed with barley squeezed after adding water and give to cattle (0.5 L/dose)	Oral	Fracture	YY39
*Croton macrostachyus* Del (Euphorbiaceae)	Bakkannisa (AO)	Leaves are pounded and squeezed with water	Nasal, topical	Bloat	YY10
*Olea capensis* L.Subsp. *macrocarpa* (C.H. Wright) I.Verd. (Oleaceae)	Woira (AM)	Leaves are chewed and applied to eye (1 teaspoon/dose)	Topical	Eye infection	YY46
*Citrus aurantifolia* (Christm.)(Rutaceae)	Lomi (AM)	Fruit is squeezed and given to hen till recovery (1/2 cup/dose)	Oral	Poultry disease	YY22
*Phytolacca dodecandra* L*.* (Phytolaccaceae)	Endod (AM)	Roots are washed and pounded; water is added and given to equine.	Nasal	Equine disease	YY09
*Calpurnia aurea* (Aiton)Benth.(Fabaceae)	Digita (AM) Ceekaa (AO)	Fresh leaves are made to paste by adding little water and rubbed to cattle	Topical nasal	Ectoparasites	YY03
*Nicotiana tabacum* L.(Solanaceae)	Tambo (AO)	Dried leaves are soaked overnight with water, squeezed and given to cattle (1 cup/dose)	Nasal	Black leg	YY13
*Withania somnifera* (L.) Dunal (Solanaceae)	Gizawa (AM)	Leaves are crushed, squeezed and given orally (2 teaspoons/dose); some leaves are smoked by placing on fire	Oral , Inhalation	Evil eye	YY24
*Justicia schimperiana* (Hochst.exNees) T.Anders (Acanthaceae)	Sensel (AM) Dhumuga (AO)	Crushing leaves, and mixing with water and drenching.	Oral	Black leg	YY30
*Ricinus communis* L.(Euphorbiaceae)	Qobo (AO)	Remove the external part of the seed, and then grind very well, mixing with water and drench the equine.	Oral	Respiratory manifestations	YY08
*Senna didymobotrya* (Fresen.) Irwin&Barneby (Fabaceae)	Sanaamaki (AO)	Grinding leaves and young stems, mixing with water and drenching (for toxin), applied topically for external parasite	Oral, topical	Snake bite, Ectoparasites	YY38
*Croton macrostachyus* Del (Euphorbiaceae)	Bakkannisa (AO)	Leaves are crushed and rubbed on infected lesion	Topical	Lesion	YY10
*Maesa lanceolata* Forssk.(Myrsinaceae)	Abayi (AO)	Leaves and seeds are pulverized and mixed with salt and water and drenched to the animal (1 L/dose)	Oral	Internal parasite	YY46
*Ensete ventricosum* (Welw.) Cheesman (Musaceae)	Qocho (AO)	Extract of the stem is applied on mouth of cattle	Topical	Foot and Mouth disease	YY05
*Dodonaea angustifolia* L.F.(Sapindaceae)	Ittacha (AO)	Leaves are pounded and mixed with salt and given to cattle	Oral	Liver disease, diarrhea	YY36
*Vernonia amygdalina* Del.(Asteraceae)	Ebicha (AO)	Leaves are mixed with salt and given to cow	Oral	Retained placenta	YY32
*Zingiber officinale* Roscoe (Zingiberaceae)	Gingibil (AM)	The dried stem is powdered and mixed with water and clear extract is applied on eye	Ophthalmic	Eye inflammation	YY51
*Ensete ventricosum* (Welw.) Cheesman (Musaceae)	Qocho (AO)	Stem is crushed and mixed with salt and given to cow	Oral	Retained placenta	YY05
*Nicotiana tabacum* L.(Solanaceae)	Tambo (AM)	Leaves are crushed, soaked in water over night, then given to cattle and sheep	Oral and nasal	Snake bite, for fattening of cattle	YY13
*Echinops kebericho* Mesfin (Asteraceae)	Kebercho (AM)	Root are powdered mixed with water and give to cattle and sheep	Oral	Skin infection	YY34

*AO*, Afan Oromo; *AM*, Amharic.

Majority of the medicinal plants reported were obtained from wild source (81.08%) and others were either cultivated (14.87%), or obtained from market (4.05%). The results of growth form analysis of the reported medicinal plant species revealed that trees constitute the largest category, with tree (43.24%), followed by shrubs (33.78%) and herbs (14.86%). Remedy preparations were made from largely leaves that accounted for (68.12%), followed by roots (14.04%), and seeds (5.8%) of the total plant parts reported. In preparation of medicine for livestock, the healers have been using various methods of preparation of traditional medicines for different types of ailments. The preparations vary based on the type of disease treated and the actual site of the ailment. The principal methods of remedy preparation from the plant parts were reported to be through concoction which accounted for 36.5%, followed by decoction (27.1%) and infusion (12.6%). The appropriate routes of administration for various diseases were used, the dominant one was oral (76.2%) followed by topical (9.53%) and nasal (5.19%). Other modes of application were also used when considered appropriate.

A very specific protocol is in use for some remedies of black leg in order to increase the effectiveness. For example, the cattle which received a remedy made from pulverizing fresh leaves of *Croton macrostachys* Del., *Calpurnia aurea* (Ait.) Benth., and *Senna didymobotrya* (fresen.) for a blackleg treatment should not graze, drink water and exposed to sunlight during the course of treatment for first two days.

### Informant consensus

Of 74 medicinal plants reported from the study area, not all were equally important. Informants frequently mentioned few medicinal plants for treatment of certain diseases. Those medicinal plants commonly reported by more informants were considered suggestive of correct remedy in our investigation activities. In this study, comparatively the highest response rate was recorded for *Croton macrostachys* Del. Fourteen respondents (16.66%) reported the use of *Croton macrostachyus* Del. for remedies against blackleg and skin infection, followed by *Nicotiana tabacum* L*.* (10.71%) and *Olea capensis* L. Subsp. *macrocarpa* (C.H. Wright) I.Verd. (9.52%) which were used against snake bite and retained placenta, respectively.

The survey additionally suggested that a greater number of species were reported to have been used as remedies for treatment of blackleg (a vernacular expression used to describe conditions presented with swelling of thigh, with legs hanged up and hair-raising which progresses to discolored, muscular damage of involved legs) which accounted for 28.23% of the total medicinal plants studied, followed by bloat (which locally expressed as the condition when the animal has abdominal distention because of gas production excessively and fluid in its stomach) (8.73%), ectoparasites (horn flies, face flies, ticks and lice which take the blood of the animal and cause blood loss, discomfort, skin injuries, hair loss, etc.) (6.79%), and respiratory manifestations (nasal discharges, sneezing, coughing, respiratory obstruction, labored breathing) (5.83%). In summary, the most common type of livestock ailments in the study area which were treated by traditional remedies are presented in Tables 1 and 2.

Result from animal health care services over past decades and future showed that 42 (50%) of the respondents opted primarily for either traditional or modern veterinary medicines based on type of disease. Some farmers, 15 (17.86%) automatically opted for traditional veterinary medicine (TVM) whereas 27 (32.14%) fully responded depending upon use of modern veterinary medicine (MVM) for their animal health care.

## Discussion

In this scientific paper, we presented an inventory property and the mode of use of plants derivatives to treat livestock ailments in four districts of Jimma zone, Ethiopia. The study showed that majority of the traditional healers were above 48 years since it is very difficult to disclose their traditional medicinal information, which they considered their indigenous knowledge as a professional secret, only to be passed orally to their eldest son in their old age.

The gender distributions of medicinal plant knowledge showed most of the traditional healers are males (94.05%) as there were very few female (5.95%) practitioners. The very good reason why greater numbers of traditional practitioner are male might be related with the local tradition of restricting such practices mostly to males whereas females were not allowed to be involved in outdoor activities but remain home as they look after babies and carry out domestic activities. It was also observed that females’ knowledge in medicinal plants is by far limited to plants, which are found in domestic environments like *Zingiber officinale* Roscoe*, Ocimum lamiifolium* Benth., *Nicotiana tabacum* L., *Lepidium sativum* L. and *Allium sativum.* Additional reason was being transfer of medicinal plant knowledge from fathers only to their first son or other male child that could keep the secret. Yirga *et al*. [28] also reported consistent result in which 100% practitioners were males and the study conducted in china by Shen *et al*. also reported consistent finding in which majority (56.7%) of traditional practitioners were males [14].

In comparison of educational status, non-educated informants handled much knowledge of traditional medicine whereas educated informants had low knowledge of traditional medicine, which is an indicative of impact of modern education.

In general, the present study identified 74 medicinal plants that have been endemically used to treat 22 different types of livestock diseases by traditional practitioners in Jimma Zone. In similar study that carried out in Southern Ethiopia among Borena pastoralists, 43 ethnoveterinary plants were documented scientifically to national ethno-botanists level [5]. Likewise, another report by Giday *et al.*[4] of study in Ofla and Raya-Azebo woredas, South Tigray also showed that they identified and scientifically documented 83 plant species for treating 37 types of livestock ailments. In same geographical region south Tigray, Seharti-Samre district, twenty two species of ethno-botanies were identified by Yirga *et al*. [20] and scientifically recognized by Ethiopian botanist that have many medicinal roles to 18 livestock ailments. Overall, these reviews of past literature show that naturally our country, Ethiopia, endowed with plenty of medicinal plants fairly distributed throughout its regions.

Our observation also agreed with medicinal plant species found elsewhere in the various study districts that are given for different diseases or have similar medicinal purposes in those regions of Ethiopia and in Africa. Among the total of Seventy-four medicinal plant species investigated in this study, 15 species in Akaki district of eastern Shewa by Bekele *et al.*[29]; 13 species in Gimbi district of Eastern Wellega by Megersa [30]; 8 species in Western Wellega by Tolossa [31]; 9 species in Goma district of Jimma zone by Behailu [25]; 8 species in Wonago district of SNNPR by Mesfin *et al*. [32], 10 species in Chelya district of Western Shewa by Amenu E. [33], 7 species in Seharti-Samre district of southern Tigray by Yirga *et al*. [20], 3 species in South Omo of SNNPR by Tolesa *et al*. [34] and 7 species in Kenya [11] were documented. All these suggest that traditional ethnoveterinary knowledge widely used in Ethiopia and there might be increases inclination of people to depend on such practices because it is considered equivalently effective to modern cares and easily available in their domestic environments.

With regard to data from the medicinal plant classes studied (Tables 1 and 2); Asteraceae was the most common plant family reported followed by Cucurbitaceae and Solanaceae. Those findings were in agreement with report by Yineger *et al.*[18] in Bale Mountains National Park in which Asteraceae as the highest, followed by Solanaceae and Fabaceae. Similarly, other researchers also stated that diversified classes of medicinal plants were identified from Asteraceae family [32].

Predominant distribution of ethno-botanical medications was commonly sought from wild sources but few were domestically cultivated as well as the remaining usually marketed. What draws greater attentions, the majority of farmers depend on wild environments where the plants are naturally grown rather than domestication of them due to beliefs about medications and promote secrecy behind such practices. It was also found from the present study area that some traditional healers did not have interest to domesticate the plant species used to treat specific ailments. The plants which were found in domestic area and had pharmacological activity are not purposely cultivated for medicinal value, but mainly for purposes like food, fence, spice and so forth. A comparable finding about distributions of the medicinal plants was also reported in many Ethiopia, African countries and Asian countries [14,20,25,31].

Among the medicinal plants found, type of them analysis showed that trees were primarily involved, followed by shrubs. The very good reason why these two plant types are the leading in medicinal practices might be attributed to their easily occurrence in the immediate environments with high level of abundance. Also the presence of more medicinal trees and shrubs in the findings indicated the fact that there might be tree and shrub rich forest resources in the area in the past. These facts might be influenced the indigenous knowledge of the local people in the area and invented as a result of frequent trial and errors. The finding of trees predominantly in medicinal purposes than other plant types for the treatment of livestock ailments is in line with research in Gimbi district, West Wellega [33]. In contrast, Kebu *et al.*[21] reported that shrubs took the leading, which accounted for nearly 45% of the medicinal plants used by Kereyu pastoralists.

Data from plant parts showed the most common phytochemical substances for medicinal purposes were frequently extracted from leaves part of ethno-botanies followed by roots and seeds, respectively. Many reports in different geographical regions of Ethiopia also consistently indicated that plant parts involved in traditional remedies formulation in this study area, almost having proportional rate of usage in Orissa, India and Faisalabad, Pakistan [4,15,19,20,22,35]. Different from our findings, a number of researchers reported that root parts were frequently involved in preparation of pharmacological substances to cure various livestock diseases occurring endemically [17,18,21,33,36]. The good reason why plant leaves most frequently used in extraction of pharmacologically active ingredients to treat diseases of livestock was, indeed, due to the fact that leaves contain many metabolites with characteristic anti-illness effects. In ecological perspective, herbal preparation that involves roots, rhizomes, bulbs, barks, stems or whole parts have effects on the survival of the mother plants [18]. Harvesting of leaves compared to harvesting of roots has a less negative influence on the survival and continuity of useful medicinal plants and hence does not affect sustainable utilization of the plants.

Analysis of mode of administration showed oral route was the most common mode of administration followed by topical and nasal route, respectively. Those findings were almost consistent with studies within different regions in Ethiopia. For instance, Teshale *et al.*[5] reported most of medicinal plants used by Borena pastoralists were administered through oral route followed by dermal route. Kebu *et al.*[21] in their study in Fentalle (Eastern Shewa) reported that greater than half of remedies studied were administered orally. A few studies, however, reported predominantly the dermal application of cultural remedies and to lesser extent oral administration [20]. They were chosen best route of administration, both oral and topical routes, because they considered permitting rapid physiological reaction with the pathogens and increasing the curative power of the medicines.

Similar to findings of previous research, preparation and application for different types of illnesses included concoction, decoction and infusion [14,36]. The most common dosage form was concoction in water followed by decoction. That was because these processes produce complete extraction of active ingredients. Plant medicines were processed mostly as mixture of two or more species in this investigation. Analysis of poly herbal prescription (Table 1) suggested that, the average number of plant species used for preparation of the remedies per prescription was found to be 3.47 (66 plant species involved in 19 prescriptions). That was due to the belief of the healers that the mixing of two or more plants has synergistic effects on the illnesses. For instance, the highest number in the poly herbal medications with admixture of two or more of them was prescribed against blackleg, the commonest disease in the study area. That observation was in agreement with the findings of study in Gimbi district, West Wellega [31] and three Nu villages of China [14]. That findings were far contrasting when compared with results of investigation in two domestic states of Ethiopia [5,21], who reported that the use of multiple plants and plant parts for single animal health problem was rare. In ways of drug formulation, majority of the farmers depend on water medium as a vehicling system, which might be due to its easily availability and universal solvent. Also additive substances like human urine, food, kerosene oil, soap and common salt were mixed in the herbal remedy preparations. The additives were used to modify flavor so that mask the taste of the medicine and ensure to intake adequate dosage of medication, which also documented in similar study in regions of Ethiopia [5,19,25,30,31,33].

Some study participants responded that restrictions are obligatory when the animal takes remedy for blackleg lest some factors always slow physiological reaction and affect the curative power of remedies. However, according to the study conducted by Tolossa in Gimbi district, no such restriction was found out in the medication of livestock [31].

Some of the medicinal plants were very popular and used widely in our study area. For instance, *Croton macrostachyus* Del*, Nicotiana tabacum* L. and *Olea capensis* L.Subsp. *macrocarpa* (C.H. Wright) I.Verd. observed as typical medicinal plants that were very common in the area with higher level of efficacy. The wide use of these medicinal plants resulted from the occurrence of the diseases to be treated by them was high why the medicinal value of the plants become a very common practices and the secrecy behind remain disclosed to every persons.

*Croton macrostachyus* Del reported in the present study for the treatment of bloat and skin lesion in cattle and also reported for the same purpose in other studies [5,25,29], indicating medicinal popularity of this species. Yirga *et al.*[28], likewise, investigated the use of this plant in combination with *Corchors depressus* for the treatment of bloat in livestock. But Tolesa E in Gimbi district of Western Wellega reported the use of the plant for the treatment of rabies [31]. Similarly, Amenu E. reported from Chelya district of Western Shewa the use of this plant for infection (mix of leaf of *Croton macrostachyus* and bulb of *Allium sativum)* and scabies (mix of leaf of *Croton macrostachyus* with *Brucea antidysenterica). *Such kind of wide spread use of a given plant species might indicate the pharmacological effectiveness [33]. *Nicotina tobacum* L. was the most frequently used plant for black leg and snake bite in the present study that also agreed with study conducted in West Wellega, Gimbi district [31].The plant product also has been used against control of ectoparasites such as leeches and ticks [4,25,29,34,37]. Tabuti *et al*. [36] in Uganda suggested the use of this plant for ophthalmic problems. However, *Olea capensis* L.Subsp. *macrocarpa* (C.H. Wright) I.Verd. was completely novel use in our study area and never ever reported in other similar investigations where the plant considered occurring endemically.

*Vernonia amygdalina* Del. was the most frequently used plant for removal of retained placenta around the study districts and also suggested in report from western Wellega region of Ethiopia and Uganda [30,36]. Ndi [38] recognized the use of this plant for antihelmenthiasis activity in Cameroon. The use of *Justicia schimperiana* (Hochst.ex Nees) T. Anders for treatment of black leg was already reported by many researchers [4,31,33].

*Senna didymobotrya* (Fresen.) Irwin & Barneby involved in treatment of venom of snake bite in livestock was already reported by Tolossa E [31]. *Citrus aurantifolia* (Christm.) for treatment of poultry disease in our study was reported by Monteiro *et al*. for the same purpose [39] and disagreed with report from study in North Western Ethiopia in which the plant products were commonly used for controlling leech infestation in livestock [40]. *Calpurnia aurea* (Aiton) Benth. for ectoparasites and black leg in our finding has also been previously documented in some areas of Ethiopia [25,30-34]. *Zingiber officinale* Roscoe for treatment of eye inflammation in our investigation that was consistently reported in previous studies [20,41]. In contrast to these findings, this plant was traditionally used against constipation in study of India [42] and black leg infections in study of Western Wellega [31]. *Lepidus sativum* L.was reported to be used against black leg that holds true in our observation [31]. *Verbascum sinaiticum* Benth. was remedy of first choice in treatment for skin infection in our study. This plant in three reports of study in Ethiopia stated its wide spread use in treatment of leech infestation, worm and *trypanosomiasis*[4,31,43]. The pharmacological activity of *Phytolacca dodecandra* L. in foot and mouth disease [31], in anthrax [4,43], in black leg [4] and in *trypanosomiasis*[43] were agreeably reported but only used against foot and mouth diseases (FMD) in this finding.

New findings of this investigation that involved in pharmacological activity against livestock diseases include plant species such as *Fagaropsis angoleusis, Allophylus macrobotryls* Gilg., *Pentas schimperiana, Olea capensis* L.Subsp. *macrocarpa* (C.H. Wright) I.Verd.*, Ekebergia capensis* Sparm.*, Leonotiso cymifolia, Gouania longispicata* Engl.*, Acmella caulirhiza* Del.*, Euphorbia schimperiana* Scheele and *Clausena anisata* Benth*.* The medicinal activity of these plants are novel findings that only known in this area for such purpose.

As suggested by our study half of the respondents stated their best choice as either TVM or MVM for animal treatment which was determined based on type of ailments. Additionally, TVM was best choice for some of respondents in animal health problems. However, majority of the respondents suggested that MVM is their first choice for livestock ailments. As a result, it was concluded that based on these findings of our study both treatment systems had higher values in health care of livestock in the area, like observation in Faisalabad district of Pakistan [15].

## Conclusions

This study suggested that farmers of four districts of Jimma zone have sound ethnoveterinary knowledge and practices. In our investigational activity, efforts made to document ethnoveterinary knowledge, practices and plants of veterinary importance. Accordingly, the study enabled us to document about 74 species of medicinal plants that have been used against 22 types of livestock diseases in study districts, so far. In general, plant parts, methods of preparation, and source of such plants were also elaboratively presented. Wild plants of veterinary importance may not be well protected, as a result can be lost due to deforestation and over grazing as they are not individual farmer’s properties. Therefore, the healers with consultation of governmental responsible ethno-botanist bodies should take care not to eradicate the medicinal plant species altogether. Awareness creation among the traditional healers and community at large are important measures to preserve their indigenous medicinal plant species [44]. Poly ethno-botanical prescriptions should be further evaluated for possible active ingredient interactions and what clinical relevancies associated with them. These indigenous knowledge and practices of four district communities should be supplemented by scientific methods to evaluate the safety, efficacy and dosage of the common medicinal plants through phytochemical and antimicrobial experimentation to determine appropriate mode of delivery, drug development and dosage in pharmacological laboratory.

## Abbreviations

TVM: Traditional veterinary medicine; MVM: Modern veterinary medicine; AM: Amharic (National Language); AO: Afan Oromo (State/Regional language).

## Competing interests

The authors declare that they have no competing interests.

## Authors’ contributions

YY designed the study, developed instruments, supervised data collection, entered and analyzed data and scientific reporting. DBH participated in instrument development, data entry, analysis, writing and revisions of the manuscript. WYA mentored study design, data collection instrument development, data analysis and manuscript writing. All authors have read and approved the final manuscript.

## Authors’ information

YY: B.Pharm., MPH, Pharmaceuticals Fund and Supply Agency, Addis Ababa, Ethiopia. DBH: B.Pharm., MSc. in Pharmacy Practice, Lecturer, Mizan-Tepi University, Mizan, Ethiopia. WAY: B.Pharm, MSc., PhD, Research and Postgraduate Program Coordinator and Assistant Professor at Jimma University, School of Pharmacy.
